# A Clinicopathological Case of Left Ventricular Assist Device Outflow Graft Stenosis

**DOI:** 10.1097/MAT.0000000000002267

**Published:** 2024-07-05

**Authors:** Yusuf Ziya Sener, Cornelis W. van der Heiden, Jelena Sjatskig, Jan von der Thüsen, Kadir Caliskan

**Affiliations:** From the * Thoraxcenter, Department of Cardiology, Cardiovascular Institute, Erasmus MC University Medical Centre Rotterdam, Rotterdam, The Netherlands; † Department of Cardiovascular Surgery, Erasmus MC University Medical Centre Rotterdam, Rotterdam, The Netherlands; ‡ Department of Pathology, Erasmus MC University Medical Centre Rotterdam, The Netherlands.

**Figure 1.**
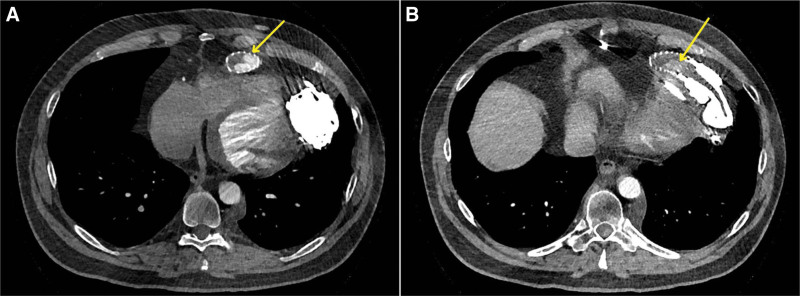
**A:** A CT angiography image showing a horizontal section of the outflow graft with stenosis due to biological material accumulation between the graft and the bend relief. **B:** Sagittal section of the outflow graft showing partial luminal stenosis. CT, Computed tomography.

**Figure 2.**
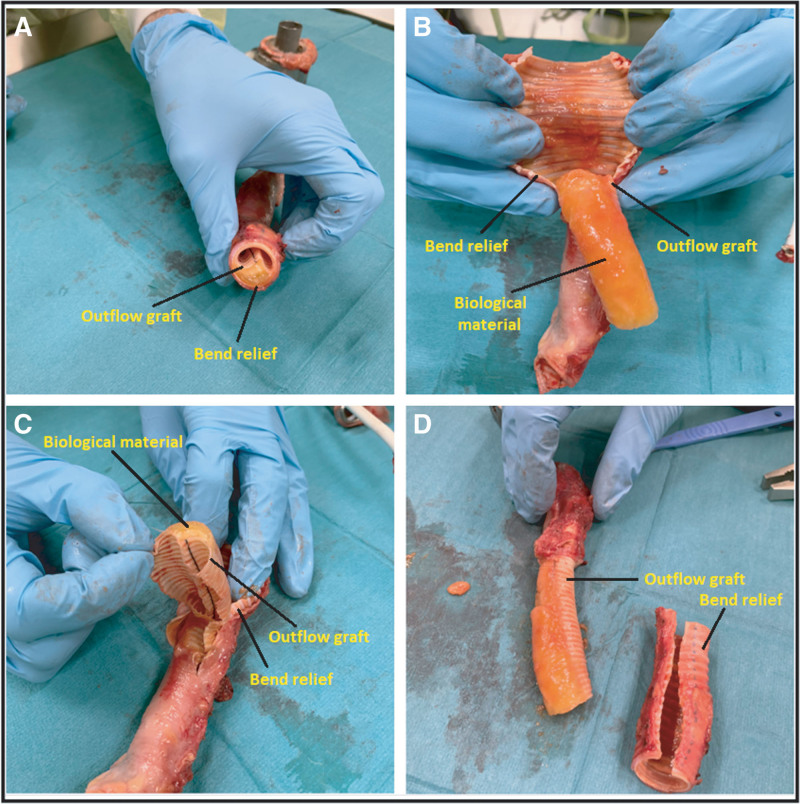
**A:** Horizontal section of the outflow graft showing accumulation of biological material between the outflow graft and the bend relief. **B:** Dissection of the bend relief shows extensive accumulation of biological material covering the graft. **C:** Both the outflow graft and the bend relief are dissected and deformation of the lumen due to external compression of the biologic material is seen. **D:** Lateral view of the graft shows the accumulated fibrofatty biologic material and aneurysmal dilatation of the bend relief at the site of the accumulated biologic material.

**Figure 3.**
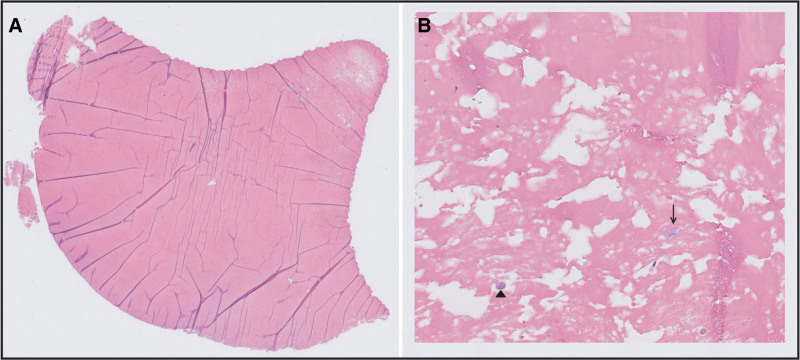
**A:** The material shows amorphous homogeneous eosinophilic staining in H&E stain and does not contain prominent aggregates of inflammatory cells. **B:** Higher magnification shows rare inflammatory cells including lymphocytes (arrowhead) and histiocytes (arrow). H&E, hematoxylin–eosin.

In 2019, a 42-year-old man underwent implantation of a HeartMate 3 left ventricular assist device (LVAD) as a bridge to transplantation for end-stage heart failure. In January 2023, he complained of occasional lightheadedness. Left ventricular assist device parameters were stable with a rate of 5,500 rpm, flow of 4.1 L/m, pulse index of 5.6, and power of 4 W. Echocardiography showed mild aortic regurgitation with stable ventricular dimensions. Computed tomography (CT) angiography was performed to evaluate the inflow and outflow graft. Outflow graft stenosis (OGS) causing approximately 45% obstruction was noted (Figure 1), probably due to external compression of the outflow graft in the “bend relief,” which is a stiff polytetrafluoroethylene covering. Given the mild severity of symptoms and normal LVAD parameters, watchful waiting was continued. CT angiography repeated 6 months later showed no change in stenosis severity. In March 2024, the patient underwent heart transplantation with LVAD explantation. Close examination of the outflow graft revealed OGS due to fibrofatty material within the bend relief (Figure 2). Microscopic evaluation of the material on hematoxylin–eosin (H&E)–stained slides was consistent with the deposition of an extensive amount of amorphous eosinophilic material without the prominent presence of inflammatory cells (Figure 3).

Outflow graft stenosis is a newly recognized serious complication of permanent LVAD support with HeartMate 3. A recent multicenter study reported an increasing incidence of OGS from 0.6% at 1 year to 9.1% at 5 years. Outflow graft stenosis may develop due to intraluminal formation of amorphous protein-like material with subsequent external obstruction, probably due to chronic organic material leaking through the graft and accumulating between the graft and the bend relief.^1^ The management approach is not yet established, but asymptomatic patients with <50% stenosis could be followed closely, while intervention should be considered in symptomatic cases with OGS >50% to 75%.^2^ Percutaneous stenting seems to be preferable to surgical approaches.^3^
